# Balanced-ternary-inspired reconfigurable vortex beams using cascaded metasurfaces

**DOI:** 10.1515/nanoph-2022-0066

**Published:** 2022-04-04

**Authors:** Ji Liu, Jurui Qi, Jin Yao, Wenman Hu, Dajun Zhang, He-Xiu Xu, Xiong Wang

**Affiliations:** School of Information Science and Technology, ShanghaiTech University, Shanghai 201210, China; Air Force Engineering University, Xi’an 710051, China

**Keywords:** balanced ternary system, broadband vortex beams, cascaded metasurfaces, high-order vortex beams, reconfigurable generation of vortex beams, vortex beams

## Abstract

Electromagnetic vortex carries the orbital angular momentum, one of the most fundamental properties of waves. The order of such vortex can be unbounded in principle, thus facilitating high-capability wave technologies for optical communications, photonic integrated circuits and others. However, it remains a key challenge to generate the high-order vortex beams in a reconfigurable, broadband and cost-effective manner. Here, inspired by the balanced-ternary concept, we demonstrate the reconfigurable generation of order-controllable vortices via cascaded *N*-layer metasurfaces. We theoretically showed that $3^{N}-1$ different vortex modes can be generated by cascading *N* metasurfaces, each one serving as an individual vortex beam generator for the order of $3^{k}$ (*k* = 0,1,2 …, N−1 ). As a proof-of-concept demonstration, a reconfigurable generation of 26 different vortex beams, with orders from 1 to 13 and from −1 to −13, is showcased in a broad millimeter-wave region by a cascade of 3 metasurfaces. Our method can be easily extended to vortex beam generator of arbitrary orders in a reconfigurable and easily implementable manner, paving a new avenue towards tremendous practical applications.

## 1 Introduction

Angular momentum is a crucial property of electromagnetic (EM) waves. Two stereotypes are majorly studied, including spin angular momentum representing two circular polarization states of EM waves and orbital angular momentum (OAM) associated with the helical phase wavefront of vortex beams [1]. An EM vortex beam of order *m* bears a spiral phase profile of exp(j*mφ*), featuring a linear proportionality of azimuth angle *φ* and an amplitude void (ring-shaped amplitude profile) at the center of field. Here, *m* denotes number of the topological charge and can take any positive or negative integer or fractional value. Vortex beams with different integer modes are orthogonal to each other, hence their unbounded topological charge *m* implies the possible multiplexing of an unlimited number of OAM beams through a single spatial aperture [2]. Accordingly, OAM as an auxiliary freedom can enable diversified fascinating applications like high-capacity information processing [3], [4], [5], [6], [7], [8], [9], [10] secure communications [11], [12], [13], object detection [14, 15], imaging [16], [17], [18] and others [19, 20].

Those intriguing applications should rely on multiplexing vortex beams with high orders, hence making highly desirable the reconfigurable, cost-effective, broadband, and easily implementable vortex beam manipulations. Several methods have been applied to generate high-order vortex beams in the microwave or millimeter-wave regime, e.g., *m* = 1, 3, 5 [21], *m* = ±1∼±6 [22], *m* = 1∼7 [23], *m* = ±1∼±9 [24], to name a few. However, as summarized in Table 1, these works are based on static devices (like circular antenna arrays [23, 25], spiral phase plates [4, 26], spiral antennas [21, 27], or metasurfaces [28], [29], [30], [31], [32], [33], [34], [35], [36], [37]) that are not tunable, implying that the order of the generated vortex beam cannot be dynamically adjusted once the device is fabricated. The programmable metasurfaces offer dynamic tunability [38], [39], [40], [41], [42], [43], [44], but exhibit several limitations such as high cost, narrow bandwidth, complex feeding circuits, insufficient binary phase tuning range, instability of the active meta-atoms, and low mode purity due to big meta-atom size. For instance, PIN-diode-based active meta-atoms only have binary phase [39]. Though varactor-based active meta-atoms can increase the phase tunability [42], they suffer from narrow-band operation and difficult fabrications. So far, only relatively low vortex orders (*m* ≤ 2) have been realized using programmable metasurfaces [38], [39], [40], [41]. In addition, PIN-diode-based active metasurfaces operating in the millimeter-wave regime are highly challenging to realize and suffer from big losses. Circular antenna arrays can also generate reconfigurable vortex beams [15], but the complicated feeding network adds complexity and cost to the system and high-order vortex beams demands a lot of antennas to launch. In the optical band, high-order vortex beams are usually launched by spiral phase plates [6, 45], metasurfaces [46], [47], [48], [49], [50], [51], and spatial light modulators (SLM) [52, 53]. However, the spiral phase plates and static metasurfaces are not reconfigurable, programmable metasurfaces are hard and expensive to fabricate, and SLMs are costly and the big element size leads to low accuracy for generating vortex beams. In brief, the reconfigurable generation of broadband vortex beams with high orders remains to be addressed, hindering the applications and advancement of vortex beams.

**Table 1:** Performance comparison between this work and previous methods for the generation of high-order vortex beams.

Methods	Reconfigurability	Phase modulation Ability	Element size	Cost and complexity	Bandwidth	Vortex order
Circular antenna arrays [23, 25]	No	Continuous	Over-wavelength	High	Broad	m=1∼7
Spiral phase plates [4, 6, 26, 45]	No	No	Over-wavelength	Low	Single frequency	Single
Spiral antennas [21, 27]	No	No	Over-wavelength	Low	Single frequency	m=1,3,5
Static metasurfaces [46, 51]	No	3 bit usually	Sub-wavelength	Low	Narrow or broad	Single or multi-order
Programmable metasurfaces [21, 38], [39], [40], [41], [42], [43], [44]	Yes	1 or 2 bit usually	Sub-wavelength	High	Narrow	m=±1∼±6
SLMs [52, 53]	Yes	8 bit usually	Over-wavelength	High	Broad	m=±1∼±12
This work	Yes	4 bit	Sub-wavelength	Low	Broad	m=±1∼±13

Here, inspired by balanced-ternary concept we propose a novel mechanism to generate reconfigurable high-order vortices in broadband frequency by cascading a few transmissive metasurface plates. The advantage of our strategy is highlighted in Table 1. The management of cascading *N* metasurfaces, each one rendering a vortex beam generator of order 3^*k*^ (*k* = 0, 1, 2, …, N−1 ), can support a vortex beam tuneable within totally $3^{N}-1$ consecutive integer vortex orders from 1 to $\sum_{0}^{N-1}3^{k}=\frac{3^{N}-1}{2}$ and from −1 to $-\frac{3^{N}-1}{2}$ , following the balanced ternary concept. For verification, we stacked three high-transmission and broadband metasurface-based vortex beam generators of order 1, 3 and 9 to launch 26 different vortex beams, with orders of 1to13 and −1 to −13. By both numerical and experimental results, we clearly demonstrate the effectiveness of the engineered metasurfaces and proposed cascading approach. The proposed technique provides a new paradigm to efficiently generate order-controllable broadband vortex beams in a reconfigurable manner and may benefit a wealth of vortex-beam-based applications.

## 2 Materials and methods

### 2.1 Balanced-ternary concept and fundamentals

**Figure 1:**
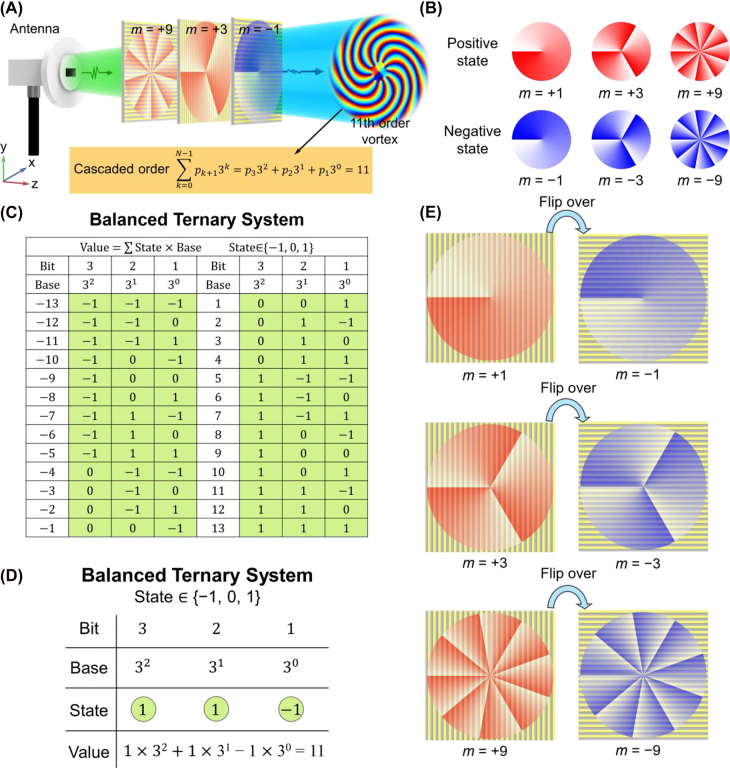
Schematic illustration of the proposed order-controllable vortices generation method based on cascaded metasurfaces. (A) Generation of an 11th order vortex beam is taken as an example in this illustration. An antenna radiates an incident EM beam. Three BVGs, i.e., order-1, order-3, and order-9, are cascaded to implement the generation of the 11th order vortex. The order-3 and order-9 BVGs are configured to generate positive-order m=+3 and m=+9 vortices in the cascading sequence, while the order-1 BVG is configured to generate a negative-order m=−1 vortex in the cascading sequence, which results in the final vortex order of $m_{f}=9+3-1=11$ . (B) Schematic transverse phase profiles of the six vortex orders used in the cascading sequence. (C) Balanced ternary system for the expression of numbers from −13 to −1 and 1 to 13. (D) is the example expressing the number 11 by the balanced ternary system. (E) Illustrations showing that flipping a transmissive-metasurface-made order-*m* BVG can generate a vortex of inverse order −*m*.

The schematic of applying cascaded metasurfaces to flexibly generate order-controllable vortex beams is illustrated in Figure 1. The basic idea is related to the balanced ternary numerical system shown in Figure 1C, which is a base-3 numerical system that expresses natural numbers by three possibilities (or states) +1, 0, and −1 in each digit with each digit representing an increasing power of 3 in decimal [54]. An ordinary ternary numerical system is also provided in Supplementary Figure S2 for comparison, which uses three states 0, 1, and 2 in each digit with each digit representing an increasing power of 3 in decimal. It is obvious that the balanced ternary system can be used to represent any integer while the ternary system can be used to represent any positive integer. Inspired by the balanced ternary system, we propose the order-controllable vortex beam generation approach by cascading in total *N* basic metasurface plates, displayed in the schematic showing in Figure 1A. To explain the idea how our design is connected with balanced ternary, we cascade a series of single metasurface plate, each one individually generating a vortex of order $m=3^{k}$ (*k* = 0, 1, 2, …, *N* – 1) in transmission and hence dubbed the order- $3^{k}$ basic vortex generator (BVG). Each involved BVG can represent a digit in the balanced ternary numerical system, with three possible states corresponding to −1, 0, and +1. Specifically, the state “+1” in the (*k* + 1)th bit corresponds to the presence of order- $3^{k}$ BVG in cascading sequence to launch $m=3^{k}$ vortex, and the state “0” in the (*k* + 1)th bit represents the absence or a void of order- $3^{k}$ BVG in the cascading. Since a flipped vortex beam generator can render the opposite vortex order [38] (as showcased in Figure 1E), the state “−1” in the (*k* + 1)th bit can be obtained via flipping the order- $3^{k}$ BVG to launch a $m=-3^{k}$ vortex. For a plane wave illumination to such cascaded BVGs, the final output beam exhibits a final vortex order *m*_f_ that is simply the summation of the orders of all the launched vortices by the cascaded BVGs, which can be expressed as

$$m_{f}=\sum_{k=0}^{N-1}p_{k+1}3^{k}$$

where *p*_*k+*1_ represents the state of the (*k* + 1)th bit that can be −1, 0, or +1. As an example, Figure 1A and D presents how to generate an 11th order vortex beam by three cascaded BVGs with orders *m* = +1, +3, and +9 and corresponding states of “−1”, “+1”, and “+1”, respectively.

Considering the fact that the balanced ternary numerical system can express any positive or negative integer number, the proposed cascaded metasurface approach can flexibly and robustly launch any arbitrary positive or negative integer order vortex, which is certainly an order-controllable and reconfigurable approach. As a consequence, the obtainable maximum positive order and minimum negative order are respectively $m=\sum_{0}^{N-1}3^{k}=\frac{3^{N}-1}{2}$ and $m=-\frac{3^{N}-1}{2}$ , which leads to a total of $3^{N}-1$ consecutive integer orders (described as ±1 to $\pm \frac{3^{N}-1}{2}$ except *m* = 0 that does not have OAM) using only *N* BVGs. The reason why we adopt the balanced ternary system rather than the ordinary ternary system, binary numerical system (base-2) or quaternary numerical system (base-4) is described in details in the Discussion section. As a proof-of-concept demonstration of the proposed order-controllable cascading vortex generation technology, we design three BVGs of orders *m* = +1, +3 and +9 to evaluate the performance. In total 26 vortex orders, i.e., ±1 to ±13, can be achieved, with the detailed cascading schemes illustrated in Supplementary Figure S1. Effectively six vortex orders given in Figure 1B contribute to the order-controllable generation of the 26 vortex beams, i.e., *m* = ±1, ±3, and ±9.

### 2.2 High-efficiency broadband meta-atom design

**Figure 2:**
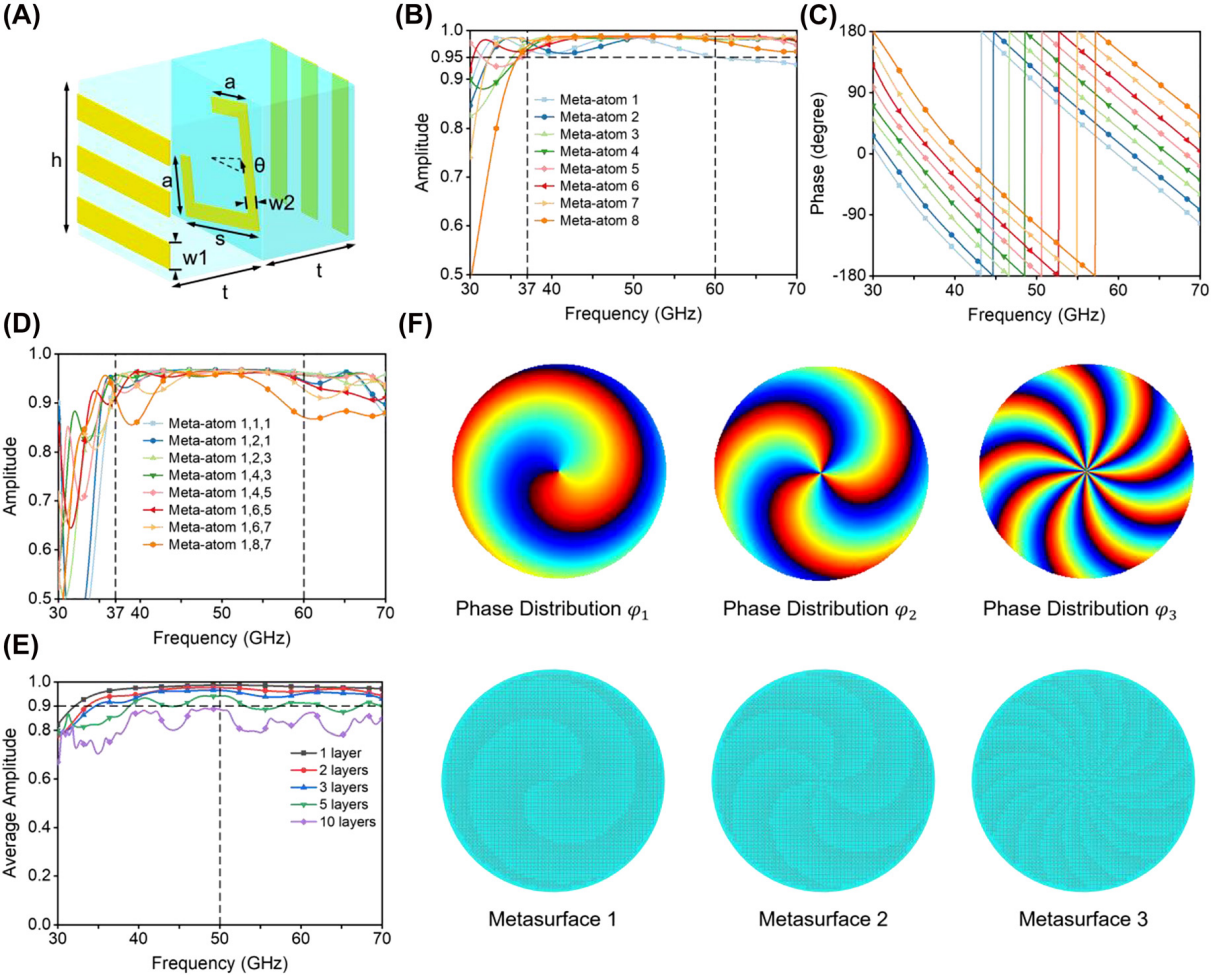
Characterization of the basic highly transmissive building block and design of metasurfaces. (A) Schematic structure of the designed broadband high-transmission meta-atom. The geometric parameters of the meta-atom are h = 1.5 mm, $w_{1}$ = 0.25 mm, $w_{2}$ = 0.15 mm, and t = 0.5 mm. (B) And (C) are, respectively, the simulated transmission amplitude and phase shift of the first 8 designed meta-atoms subject to an x-polarized incident wave. (D) Simulated transmission amplitude of different combinations of three layers of cascaded meta-atoms. (E) Simulated transmission amplitude of 1, 2, 3, 5 and 10 layers of cascaded meta-atoms. Each spectrum is the average of 8 cases using meta-atoms 1 to 8 and the same meta-atoms are used in the cascading sequence. (F) Phase distributions of three BVGs (1st row) and middle gapped-ring layers (2nd row) of the three engineered metasurfaces.

The proposed mechanism is based on cascaded transmissive metasurfaces with broadband feature. Such cascaded metasurface system can offer new freedoms to manipulate the light, both in near field [55]and far field [56], significantly enriching the functionality of metadevices. Considering the multiple metasurface-based BVGs as shown in Figure 1A, the meta-atoms need to have very high transmission efficiency to avoid severe attenuation caused by transition and multiple reflections between BVGs, which can maintain the high-efficiency and high-quality of the final vortex beam. The designed meta-atom is composed of three layers with metallic structures separated by two layers of low-loss F4B substrate (*ε*_r_ = 2.2, tan*δ* = 0.002) as shown in Figure 2A. The top and bottom metallic layers are gratings arranged to be perpendicular to each other to form a Fabry–Perot-like resonant cavity, which can dramatically ameliorate the transmittance of the meta-atom [57]. The middle metallic layer is a gapped rectangular ring that controls the phase of transmission. Both the gratings and gapped metallic ring can operate in a broad frequency range [32]. Detailed working mechanism of the Fabry–Perot-like resonant cavity is provided in Supplementary Note S2 and Supplementary Figure S3. Through adjusting dimensions and rotation angle of the gapped ring, the meta-atom can effectively achieve full 2*π* phase control in a broad bandwidth. The orthogonal grating layers ensure the incident and transmission waves of a single meta-atom are both linearly polarized and always perpendicular to each other. Varying the thickness of the F4B substrate layer can shift the operating frequency band of the meta-atom, which is discussed in Supplementary Note S10.

In order to achieve precise control of the EM wave, we design 16 meta-atoms by controlling *a*, *s* and *θ* to obtain the full 2*π* phase tuning in a discrete manner. It is found that using 16 meta-atoms to build the BVGs can lead to better vortex mode purity than only using 8 meta-atoms, especially for vortex modes that need multiple cascaded BVGs to generate. The corresponding optimized parameters at 50 GHz are summarized in Supplementary Table S1. The meta-atoms 9 to 16 are simply attained by rotating the meta-atoms 1 to 8 by 90° counter-clockwise, which results in the same transmission amplitude but a phase increment of *π* compared to the meta-atoms 1 to 8. The simulated results of the meta-atoms 1 to 8 given in Figure 2B suggest that the transmission amplitude of all the meta-atoms is above 0.95 from 37 to 60 GHz and the average transmission amplitude at 50 GHz is 0.99. The simulated phase shifts in Figure 2C showcase that the phase increments of any two adjacent meta-atoms are well maintained around *π*/8 and the phase coverage of 2*π* is achieved with good broadband performance. The simulation results prove that the high-transmission broadband meta-atoms can be used to construct the high-transmission BVGs.

After elucidating the working principle of a single meta-atom, we further explore the operating mechanism of cascaded meta-atoms. Since a single meta-atom is endowed with high transmission efficiency in a broad band, the transmission efficiency of two or three cascaded meta-atoms can still be kept at a favourable level, which is proved by the simulated transmission amplitude of different combinations of three cascaded meta-atoms in Figure 2D. Most of the tested cases result in amplitude higher than 0.9 in the entire design bandwidth. We further obtain the average transmission amplitude of 1, 2, 3, 5, and 10 cascaded meta-atoms (average of meta-atoms 1 to 8 and the same meta-atoms are used in the cascading sequence) and plot the results in Figure 2E. Even 10 layers of meta-atoms can still maintain an average transmission amplitude of above 0.8, shedding light on the future applications of the proposed method for generating even higher order vortex beams by cascading more metasurface-based BVGs.

### 2.3 Vortices generator design and numerical results

We use the designed 16 high-transmission meta-atoms to construct the three BVGs of orders *m* = +1, +3, and +9, with helical azimuthal phase dependence of exp(j*φ*), exp(j3*φ*), and exp(j9*φ*), respectively. Phase profile of a convex lens (*f* = 200 mm at 50 GHz) is superimposed to the helical phase distribution of each BVG to counteract the beam diffraction. The designed phase distributions of the three BVGs and the corresponding middle gapped-ring layer of engineered metasurfaces are displayed in Figure 2F. Each metasurface is composed of 60 × 60 meta-atoms.

**Figure 3:**
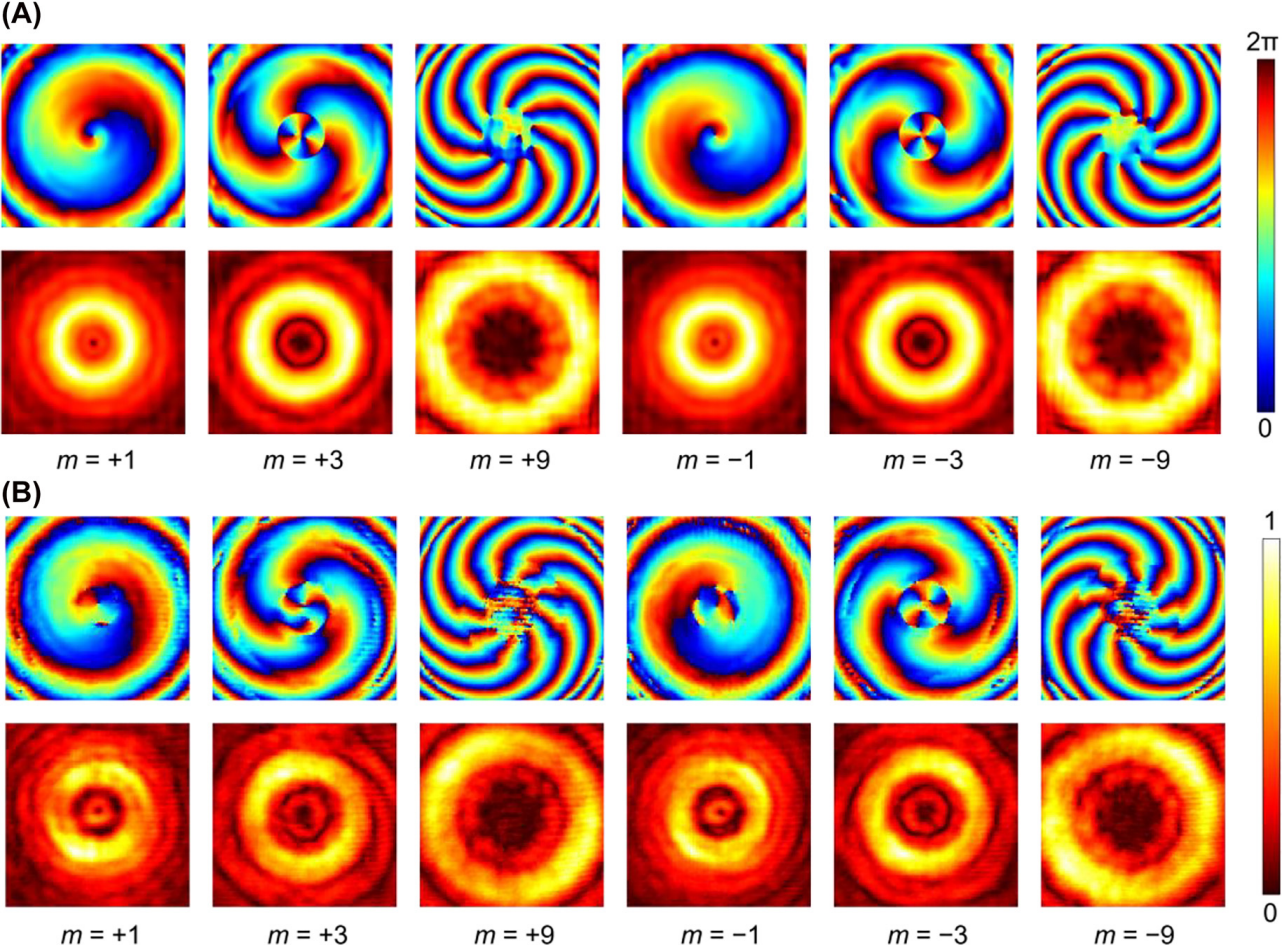
Numerical and experimental characterization of the three BVGs individually. (A) Numerically calculated and (B) experimentally measured phase profiles and amplitude distributions at 50 GHz in six cases with m=±1 , ±3 and ±9 obtained using three individual BVGs without cascading. The phase profiles are plotted in jet colorbar and the amplitude distributions are plotted in hot colorbar.

**Figure 4:**
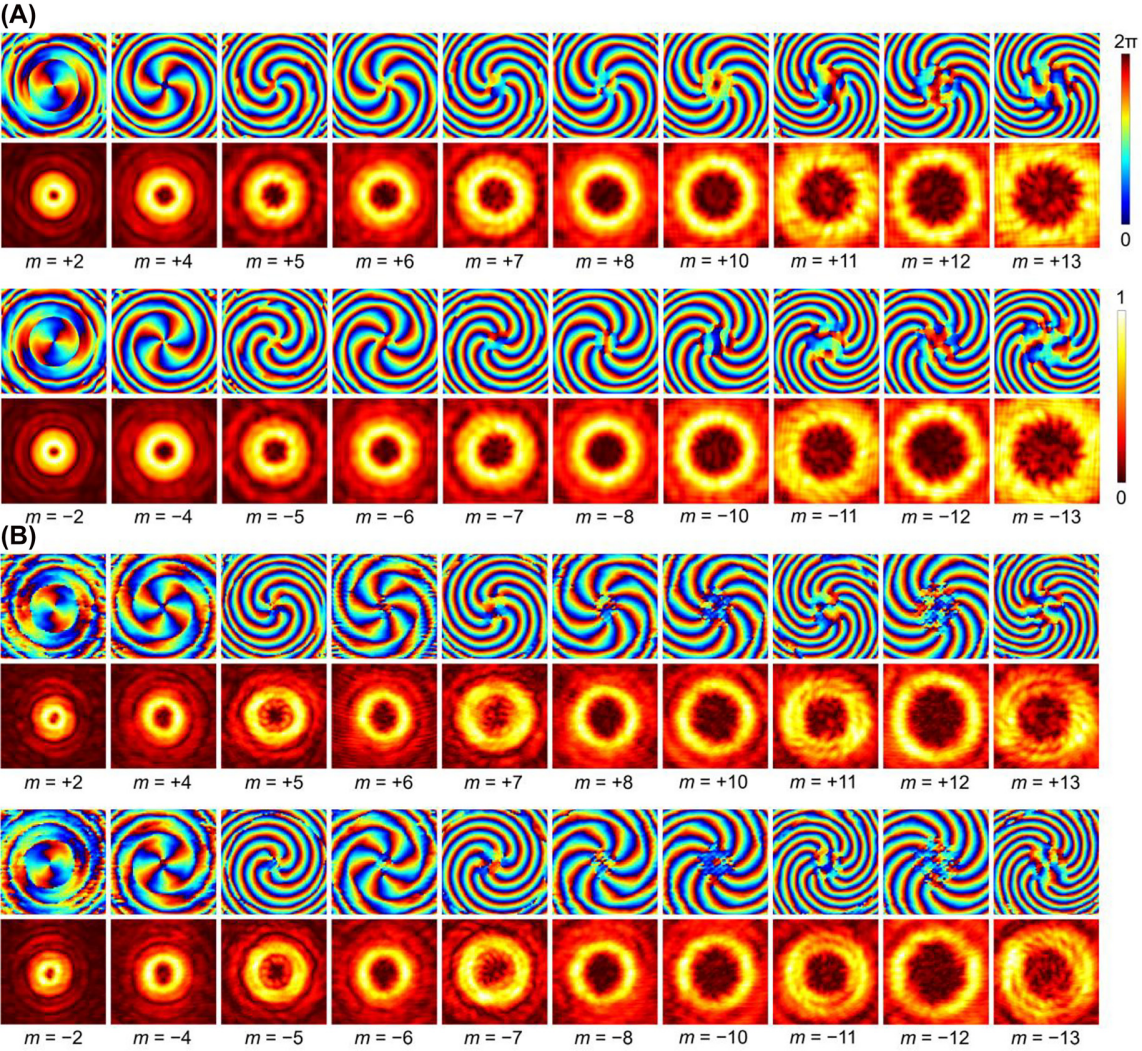
Obtained phase and amplitude results of 20 vortex modes at 50 GHz implemented by cascading the three engineered BVGs according to the method given in Figure S1. (A) Simulated and (B) experimentally measured phase profiles and amplitude distributions. The phase profiles are plotted in jet colorbar and the amplitude distributions are plotted in hot colorbar.

We perform full-wave simulations in CST to test the three BVGs. Each BVG is individually simulated with two configurations, with one representing the “+1” state and the other embodying the “−1” state by flipping over the BVG. The illumination beam is a linearly polarized source provided by a WR-22 waveguide antenna 150 mm away from the BVG. Figure 3A shows the simulation results at 50 GHz extracted on a plane 150 mm behind the BVG. The obviously observed spiral phase profile, ring-shaped amplitude null, and high-purity vortex mode spectrum (see Supplementary Figure S4) indicate reliable generation of vortex beams with modes *m* = ±1, ±3, and ±9 by the three BVGs. In addition, the results also verify that the cascading of the BVGs works well at 40 and 60 GHz (see Supplementary Figure S5), which demonstrates the good broadband characteristic of the metasurfaces that is meaningful for many applications of vortex beams. The simulation results of the rest 20 vortex orders besides those in Figure 3A can be found in Figure 4A.

## 3 Results

To validate the effectiveness of the cascaded metasurfaces for generating order-controllable vortex beams in a reconfigurable manner, the three engineered BVGs are fabricated by printed circuit boards and experimentally characterized employing the near-field scanning technique. Each of the three fabricated metasurface-based BVG consists of 80×80 meta-atoms that translates to an overall size of 120mm × 120 mm. The complete experimental setup is given in Supplementary Note S6. All the 26 vortex orders, i.e., ±1 to ±13, are experimentally lunched and detected. Measured results of the generated vortex beams with modes *m* = ±1, ±3, and ±9 using a single BVG are provided in Figure 3B, which exhibit high quality and are in very good accordance with the simulation in Figure 3A.

**Figure 5:**
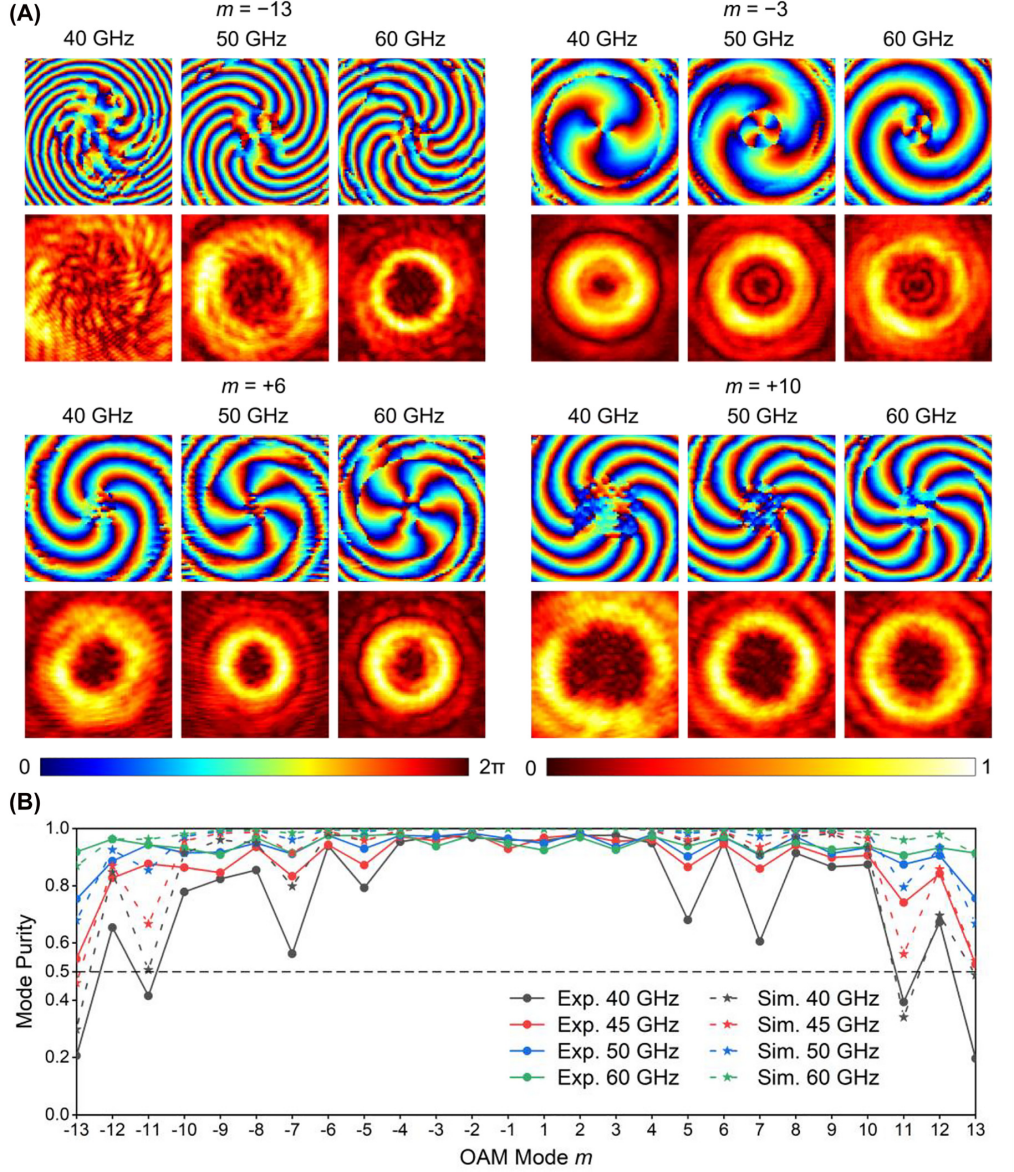
Broadband results of the proposed method based on cascading three BVGs. (A) Experimentally measured phase profiles and intensity distributions at 40, 50, and 60 GHz, where four typical vortex modes are presented. The phase profiles are plotted in jet colorbar and the amplitude distributions are plotted in hot colorbar. (B) Mode purity of the 26 vortex modes at different frequencies obtained by simulation and experimental results.

Figure 4 gives the simulated and measured phase and intensity distribution of 20 vortex modes by cascading the three BVGs making use of the protocol shown in Supplementary Figure S1. It is observed that spiral phase profiles with correct number of arms and excellent ring-shaped amplitude distributions are manifested in all tested cases. Figure 5A displays the experimental results for some modes generated at 40, 50, and 60 GHz, including the cases of a single BVG as well as cascading of two and three BVGs. The results for different modes at different frequencies show good performance, except that the performance of *m* = −13 mode at 40 GHz is slightly degraded in terms of the quality of the phase profile. The mode purity [28] at different frequencies is plotted in Figure 5B. As illustrated, the mode purity of all 26 generated vortex modes is above 0.78 at 50 GHz, indicating the high quality of the produced vortex beams. Besides, the purity of all the modes is still very high at 60 GHz, while the purity of some modes obviously drops at 40 GHz. This is mainly attributed to the fact that the phase shift of the designed meta-atoms at 40 GHz is somewhat worse than that at higher frequencies. Moreover, because the cascading of three metasurface-based BVGs tends to induce more errors (such as alignment of the BVGs in the experimental system and multiple reflections between BVGs) and suffer from more distortions during the wave propagation. Since the mode purity of vortex beams is a critical feature for practical applications, we can redefine the bandwidth of the proposed approach based on the measured results. Under the criterion of purity above 0.5, the bandwidth should be 45–60 GHz, still suggesting a very broadband operation. To sum up, all presented experimental results undoubtedly validate the effectiveness and high efficiency of the reconfigurable generation of order-controllable vortex beams. Besides the mode purity, we also investigate the absolute efficiency of the generated vortex modes, which is given in Supplementary Note S8.

## 4 Discussion

Although only three BVGs are cascaded in the presented proof-of-concept demonstration, the proposed strategy essentially works for any number of BVGs. If much smaller meta-atoms can be designed in the targeted frequency band, order-27 BVG is likely to be achieved and orders ±1 to ±40 can be obtained. Furthermore, the efficiency of applying only a few BVGs to generate a large number of order-controllable vortex beams is even higher if more BVGs are used. Such efficiency can be defined as the ratio of the total number of obtainable vortex orders to the number of BVGs, i.e., $\frac{3^{N}-1}{N}$ , which increases exponentially as *N* increases.

Since our reconfigurable vortices generation technique is inspired by the balanced ternary concept, the applied BVG needs to have three different tuneable states to represent the three possibilities +1, 0, and −1. The transmissive metasurface-based BVGs can be made present, absent or flipped in the cascading sequence to felicitously realize the required three states. However, flipping over a BVG may not be possible in some scenarios, which means only two tuneable states exist. In such cases, a binary numerical system (base-2) has to be used to generate consecutive order-controllable vortex beams and only positive orders can be obtained, e.g., applying BVGs with order of $m=2^{k}$ (*k* = 0, 1, 2, …, *N* – 1). But this binary-system-based method has an efficiency of $\frac{2^{N}-1}{N}$ , which is not as efficient as the balanced ternary system method. Similarly, the ordinary ternary system or other numerical systems, e.g., quaternary numerical system (base-4), can only generate positive vortex orders. As suggested by Supplementary Figure S2, the ordinary ternary system requires the states of “+1” and “+2”, which can be implemented by BVGs of order $m=3^{k}$ and $m=2\times 3^{k}$ , i.e., m=1,2,3,6,9,18,… . It should be noted that only using BVGs of order $m=3^{k}$ cannot generate consecutive vortex orders. Therefore, *N* BVGs can generate a maximum of $\frac{3\left(3^{N/2}-1\right)}{2}$ different vortices, with *N* being an even number. The resulting efficiency is $\frac{3\left(3^{N/2}-1\right)}{2N}$ . For the quaternary numerical system, *N* BVGs ( $m=4^{k}$ and $m=2\times 4^{\text{k}}$ , i.e., 1, 2, 4, 8, 16, 32, …) can generate a maximum of $2^{N}-1$ different vortices, with *N* being an even number, which is essentially identical to the binary system. The resulting efficiency is $\frac{2^{N}-1}{N}$ , which is the same as the binary system. Therefore, it is obvious that the applied balanced ternary system has the highest efficiency among all the numerical systems for generating consecutive vortex order.

As suggested by the simulated very high transmission efficiency of the metasurface plates, the multiple reflections between the cascaded BVG plates is very weak and does not affect the results too much. But losses in the metals and dielectric substrate of the fabricated BVGs cannot be avoided, which can cause some multiple reflections between the BVGs and degrade the quality and absolute efficiency of the generated vortex beams.

Besides the normal incidence used in the simulation and experiments, it is very necessary to study the influence of oblique incidence. Detailed setup and results are given in Supplementary Note S9. It is observed that a small incident angle of 5° or 10° can still render good quality of the generated vortex beams, which is acceptable in practical applications. Thus, it is concluded that the tolerable incident angle is about 10° for the proposed technique.

The proposed approach is also valid for other EM spectrum, including visible frequency. So long as flipping over transmissive metasurfaces or other kinds of transmissive vortex generators can give the opposite vortex orders, the proposed method can be directly applied in any band [57], [58], [59]. For some special optical transmissive vortex generators that uses opposite circular polarized illumination beams to generate opposite vortex orders [60], it is still possible to implement the proposed technique by properly designing the setup.

## 5 Conclusions

In summary, we have proposed an efficient method to realize reconfigurable broadband order-controllable generation of massive vortex beams by cascaded transmissive metasurfaces. This new technique is inspired by the balanced ternary numerical system and can obtain any positive or negative vortex order in a reconfigurable manner. This is a very efficient vortex generation protocol that outperforms its counterparts in terms of reconfigurability, bandwidth, cost efficiency, and quality of generated vortex beams. To demonstrate the proposed method, we desire high transmission efficiency broadband meta-atoms operating in the millimeter-wave range and construct three metasurface-based BVGs of orders +1, +3, and +9 to generate vortex beams with orders of ±1 to ±13. Both the simulations and experimental results verify the effectiveness of the cascaded metasurface concept. This novel approach can be extended to terahertz or optical bands and has great potentials in high-capacity wireless communications, imaging, and other related applications.

## Supplementary Material

Supplementary Material
