# Supplementary material for: Balanced-ternary-inspired reconfigurable vortex beams using cascaded metasurfaces
Source: Nanophotonics. 2022 Apr 4;11(10):2369–79. doi: 10.1515/nanoph-2022-0066 (PMC11636462; doi:10.1515/nanoph-2022-0066)
Supplement: Supplementary file 1 — Supplementary Material [file j_nanoph-2022-0066_suppl.pdf]

Supplementary

Ji Liu[a], Jurui Qi[a], Jin Yao, Wenman Hu, Dajun Zhang, He-Xiu Xu*, Xiong Wang*

Balanced-Ternary-Inspired Reconfigurable Vortex Beams Using Cascaded Metasurfaces

Supplementary Note S1. Working principle of balanced ternary system

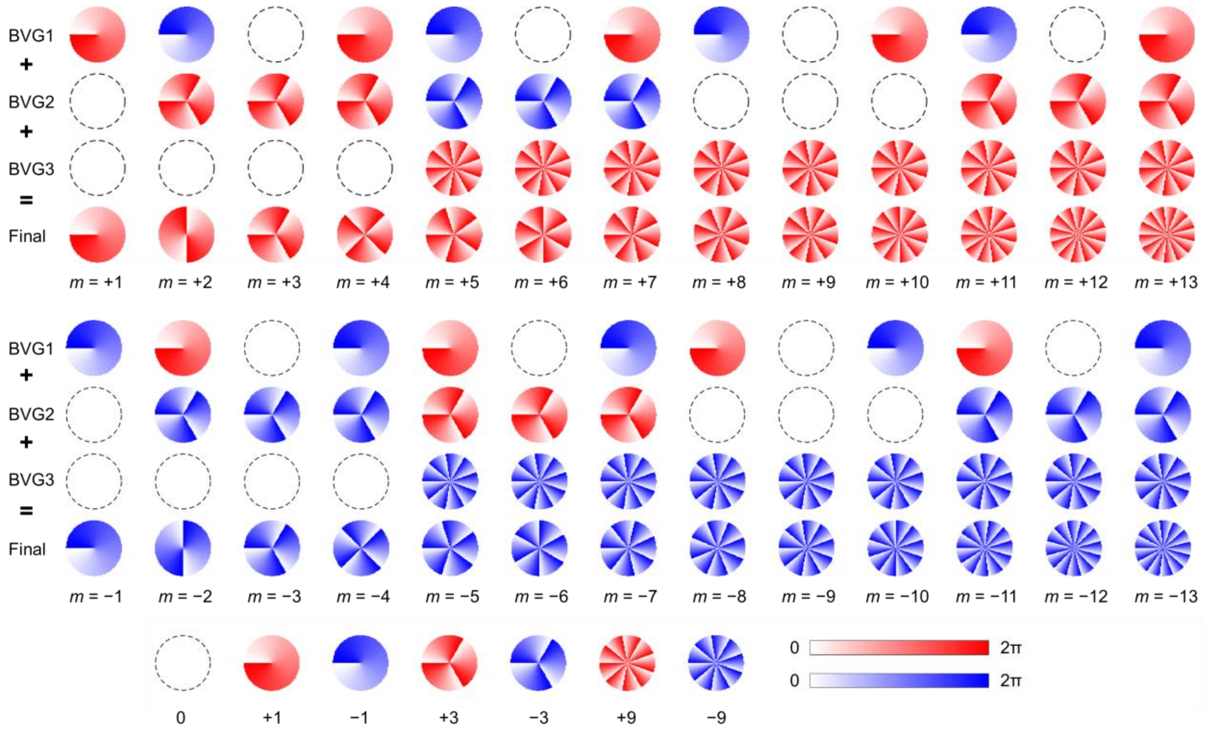

Supplementary Figure S1. Cascading schemes for generating 1 to 13 and −1 to −13 vortices by three BVGs of orders m = +1, +3 and +9. The legend is provided at the bottom. The red phase profiles represent positive vortex orders and blue ones represent negative vortex orders. The dashed circle represents the state “0” or a void bit.

(a) Ternary System

Value = $\sum$ State × Base				State∈{0, 1, 2}			
Bit	3	2	1	Bit	3	2	1
Base	3^2	3^1	3^0	Base	3^2	3^1	3^0
1	0	0	1	14	1	1	2
2	0	0	2	15	1	2	0
3	0	1	0	16	1	2	1
4	0	1	1	17	1	2	2
5	0	1	2	18	2	0	0
6	0	2	0	19	2	0	1
7	0	2	1	20	2	0	2
8	0	2	2	21	2	1	0
9	1	0	0	22	2	1	1
10	1	0	1	23	2	1	2
11	1	0	2	24	2	2	0
12	1	1	0	25	2	2	1
13	1	1	1	26	2	2	2

(b) Ternary System
State ∈ {0, 1, 2}

Bit	3	2	1
Base	3^2	3^1	3^0
State	1	0	2
Value	$1 \times 3^2 + 0 \times 3^1 + 2 \times 3^0 = 11$		

Supplementary Figure S2. (a) Ternary system for the expression of numbers from 1 to 26. (b) An example expressing the number 11 by the ternary system.

Supplementary Note S2. Working principle of Fabry-Perot-like resonant cavity

The underlying mechanism is that the two orthogonal grating layers and middle gapped ring constitute the Fabry-Perot-like resonant cavity enhancing the polarization conversion efficiency. The gapped metallic ring can induce electric dipoles in both *x* and *y* directions for polarization conversion of the transmitted EM waves. As shown in Figure S3a, an *x*-polarized incident wave E_x^{i1} transmits the front grating layer without disturbance and then interferes with the gapped ring via induced currents on the ring. The gapped ring converts a portion of E_x^{i1} into *y*-polarized transmitted and reflected waves. The *y*-polarized transmitted component E_y^{t1} can directly transmit the back grating, while the *x*-polarized component is completely reflected back from the back grating to interfere with the gapped ring again and regenerates *y*-polarized transmitted component E_y^{t2}. The *y*-polarized reflected component is completely blocked by the front grating, causing multiple reflections in the cavity and an eventual *y*-polarized wave E_y^{t3} escapes the back grating. Thickness *t* of the substrate is chosen to be 0.5 mm so that E_y^{t1}, E_y^{t2} and E_y^{t3} can be made largely in phase. Therefore, multiple reflections and polarization conversions take place in the substrates of the meta-atom effectively render high polarization conversion efficiency and transmittance owing to the Fabry-Perot-like cavity.

As provided in Figure S3b, two meta-atoms are cascaded with a separation distance *d* = 1.5 mm. The back grating of the meta-atom 1 and front grating of the meta-atom 2 have to be aligned in the same direction to accommodate wave transmission. The *y*-polarized transmitted component E_y^{i2}, which is the superposition of E_y^{t1}, E_y^{t2} and E_y^{t3}, can transmit the front grating of the meta-atom 2. With the same polarization conversion processes mentioned above, the *x*-polarized transmitted components E_x^{t1}, E_x^{t2} and E_x^{t3} can escape the meta-atom 2. The structure and polarization conversion process of the three cascaded meta-atoms is displayed in Figure S3c.

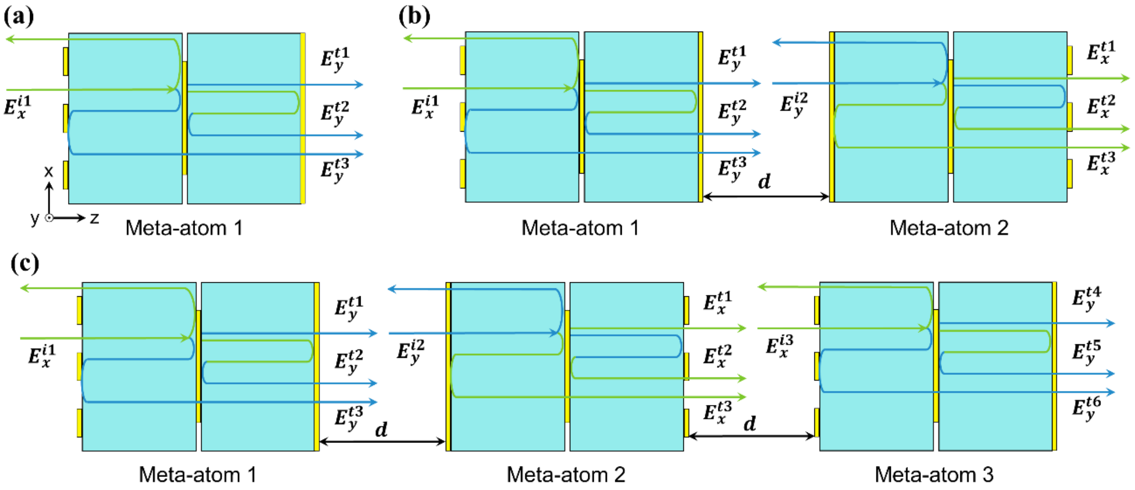

Supplementary Figure S3. Working principle of Fabry-Perot-like resonant cavity. (a), (b) and (c) respectively illustrates the wave propagation and polarization conversion process in a single meta-atom, cascaded two meta-atoms and cascaded three meta-atoms. The green rays represent *x*-polarized waves and the blue rays represent *y*-polarized waves.

Supplementary Note S3. Design parameters of the meta-atoms

Table S1. Detailed geometrical parameters and corresponding transmission amplitude and phase shift of the 16 designed meta-atoms at 50 GHz.

Meta-atom	a [mm]	s [mm]	θ [degree]	Amplitude	Phase [degree]
1	1.01	1.26	0	0.986	0
2	0.92	1.25	0	0.984	18.58
3	0.82	1.20	0	0.988	39.71
4	0.70	1.20	0	0.985	61.82
5	0.58	1.20	0	0.985	84.23
6	0.46	1.20	0	0.988	106.69
7	0.30	1.22	0	0.989	129.37
8	0.15	1.22	0	0.989	150.70
9	1.01	1.26	90	0.986	180.12
10	0.92	1.25	90	0.984	197.54
11	0.82	1.20	90	0.988	219.71
12	0.70	1.20	90	0.985	241.86
13	0.58	1.20	90	0.985	264.38
14	0.46	1.20	90	0.988	284.94
15	0.30	1.22	90	0.989	309.32
16	0.15	1.22	90	0.989	333.90

Supplementary Note S4. Mode spectrum of simulation and experimental results

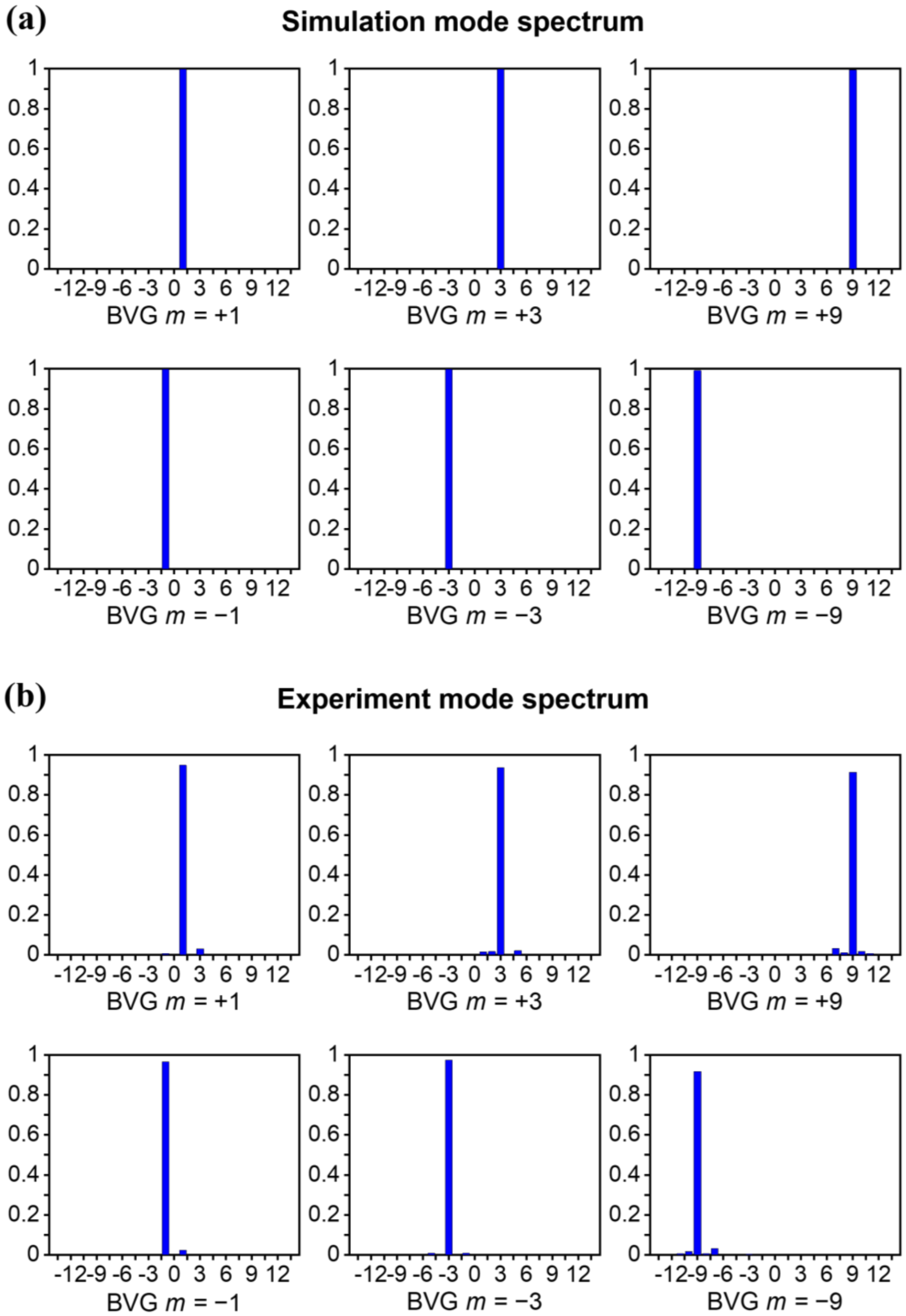

Supplementary Figure S4. Numerically calculated (a) and experimentally measured (b) vortex mode spectrum at 50 GHz in six cases with $m = \pm 1$, ± 3 and ± 9 obtained using three individual BVGs without cascading.

Supplementary Note S5. Broadband simulation results

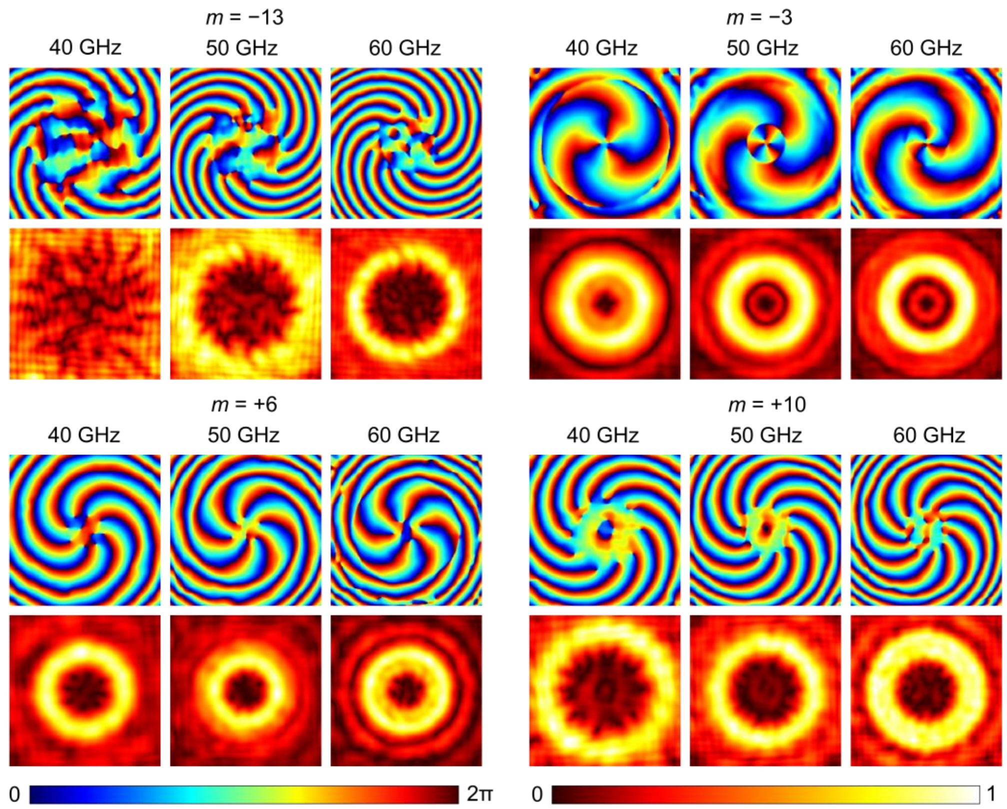

Supplementary Figure S5. Simulated phase profiles and amplitude distributions at 40, 50 and 60 GHz of four typical vortex modes.

Supplementary Note S6. Experimental setup and errors in the experiments

A pair of WR-22 waveguide antenna is used to preform measurement from 40 to 60 GHz, as shown in Figure S3a. The transmitting antenna is 150 mm in front of the cascaded BVGs and the receiving antenna is 200 mm behind the cascaded BVGs. The receiving antenna is connected to a motorized stage for scanning a planar surface with the overall range of 200 mm × 200 mm. The two antennas are connected to the two ports of a PNA network analyzer (Keysight N5227A). Both the transmitting and receiving antennas are linearly polarized and the relationship between their polarization, i.e., parallel or orthogonal, is determined by the number of cascaded BVGs in each tested case. For example, odd number of BVGs require orthogonal polarization between the transmitting and receving antennas, while even number of BVGs require parallel polarization. We use rigid customized metal frames (shown in Figure S5e) to mount the cascaded BVG plates and apply screws at the corners of all the BVG plates, which can guarantee very accurate alignment.

It is seen that there are some differences between the simulated and measured results in Figure 3, especially the measured ring-shaped amplitude distributions are not as uniform as the simulated ones. Such discrepancy between the simulated and measured results is probably due to some errors in the experiments. First, the overall thickness of each BVG metasurface plate is only 1 mm and the applied two 0.5-mm-thick F4B dielectric substrate material is not rigid enough. So, the BVG plates are bent a little bit even if they are mounted on the metal frame using screws. The resulting curvature leads to a non-uniform distribution of the electric fields passing through the cascaded BVGs. Increasing the substrate thickness or using a more rigid substrate material may alleviate this

problem. Second, the motorized stage used in the experiments is not covered by absorbing materials, which is also very hard to do. This can induce some reflections between the BVG plates and the motorized stage, which adversely affects the measured amplitude and phase results and the associated absolute efficiency.

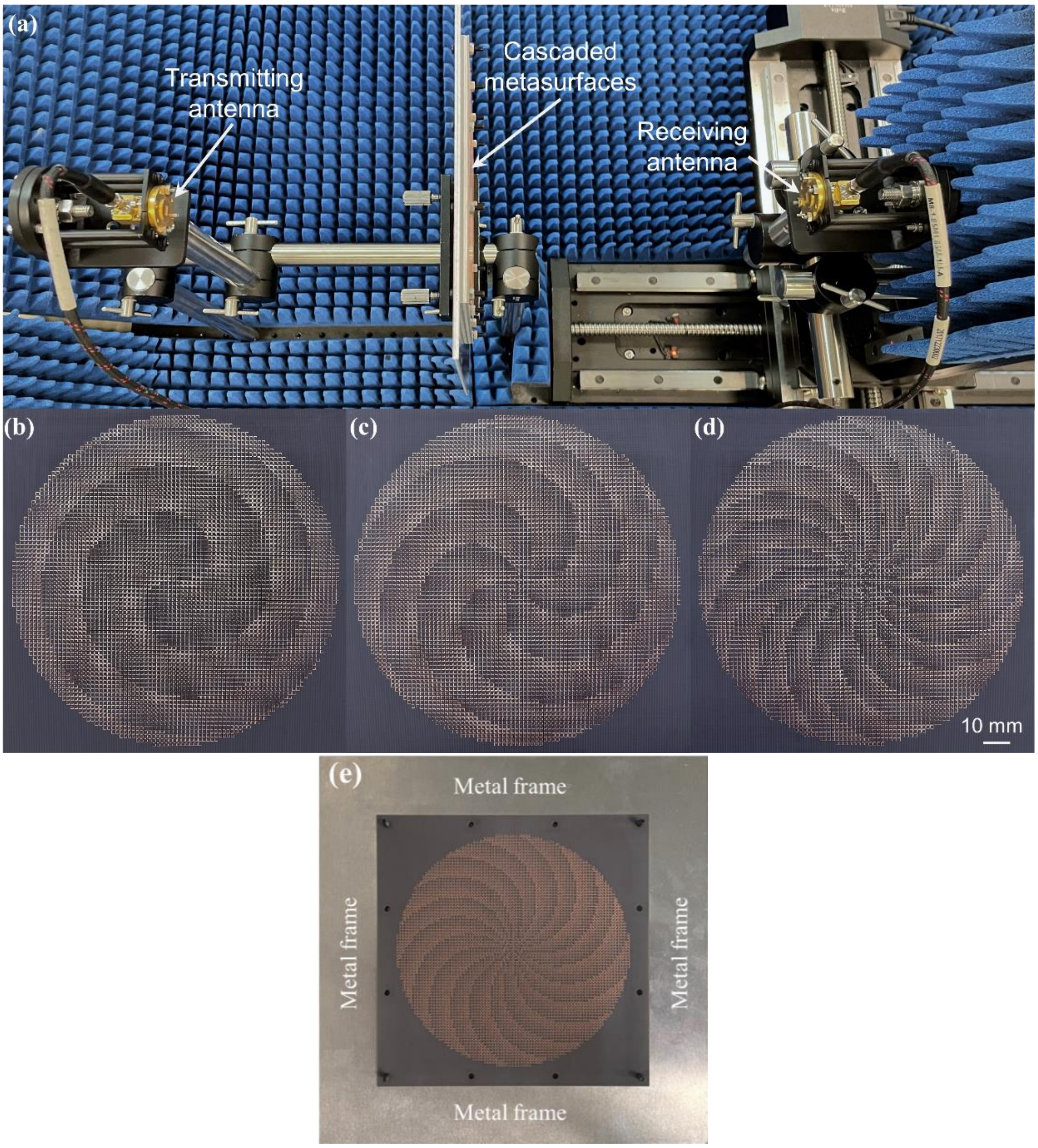

Supplementary Figure S6. Experimental setup measuring the generated vortex beams by the three BVGs. (a) Photo of the experiment setup of the near-field scanning technique. The transmitting and receiving antennas are connected to a network analyzer. (b)-(d) are the photos of the fabricated middle gapped-ring layers of the three engineered BVGs. (e) Photo of a BVG mounted in a square metal frame.

Supplementary Note S7. Efficiency comparison of different numerical systems

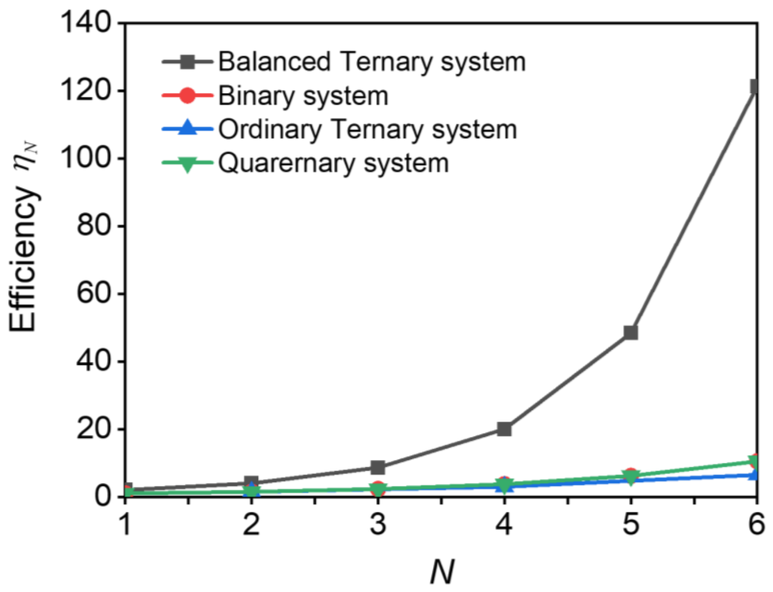

Supplementary Figure S7. The effienciency of different numerical systems. The efficiency of the binary system and quarenary system are the same.

Supplementary Note S8. Absolute efficiency of the generated vortex beams

We have obtained the absolute efficiency of the generated vortex beams for both the simulated and measured cases. The absolute efficiency η_a is calculated based on the obtained fields in a xxxxx mm × xxxxx mm region parallel to and 15 mm away from the cascaded BVG metasurfaces. Considering the polarization transformation of the metasurface, when the number of cascaded BVGs is 1 or 3, the absolute efficiency is defined as

$$\eta_a = \frac{\int E_{tx}{}^2 ds}{\int E_{oy}{}^2 ds} \times 100\%$$

where E_{tx} is the measured *x*-polarized electric field traversing the cascaded BVGs and E_{oy} is measured *y*-polarized electric field with the metasurface absent. Both the integrations are performed in the xxxxx mm × xxxxx mm region. If the number of cascaded BVGs is 2, the absolute efficiency is defined as

$$\eta_a = \frac{\int E_{ty}{}^2 ds}{\int E_{oy}{}^2 ds} \times 100\%$$

where E_{ty} is the measured *y*-polarized electric field traversing the cascaded BVGs.

The calculated absolute efficiency is listed in Table S2 in the Supplementary Note S8. It is seen that the simulated absolute efficiency for a single BVG is over 87% for all the cases across the entire bandwidth. The measured absolute efficiency is over 75% for most of the cases across the entire bandwidth. For the cascaded BVGs, the absolute efficiency generally drops. It is expected that the absolute efficiency at the boundary of the operating band is lower than that at 50 GHz (over 82% for all the cases), which is because the meta-atoms have the best performance at 50 GHz.

Table S2. Absolute efficiency η_a of different vortex orders.

Efficiency η_a (%)	40 GHz (Sim.)	50 GHz (Sim.)	60 GHz (Sim.)	40 GHz (Exp.)	50 GHz (Exp.)	60 GHz (Exp.)
m = +1	96.39	94.66	93.87	91.82	95.57	62.96
m = +3	95.36	94.28	93.63	83.26	92.00	88.72
m = +4	86.08	87.27	85.56	64.85	90.68	68.88
m = +5	74.06	83.01	83.68	50.46	90.60	77.31
m = +6	84.31	83.45	84.99	68.93	94.36	91.12
m = +8	82.40	82.56	86.07	79.80	91.60	90.51
m = +9	87.35	91.08	90.18	75.13	96.11	85.06

Supplementary Note S9. Study of oblique incidence

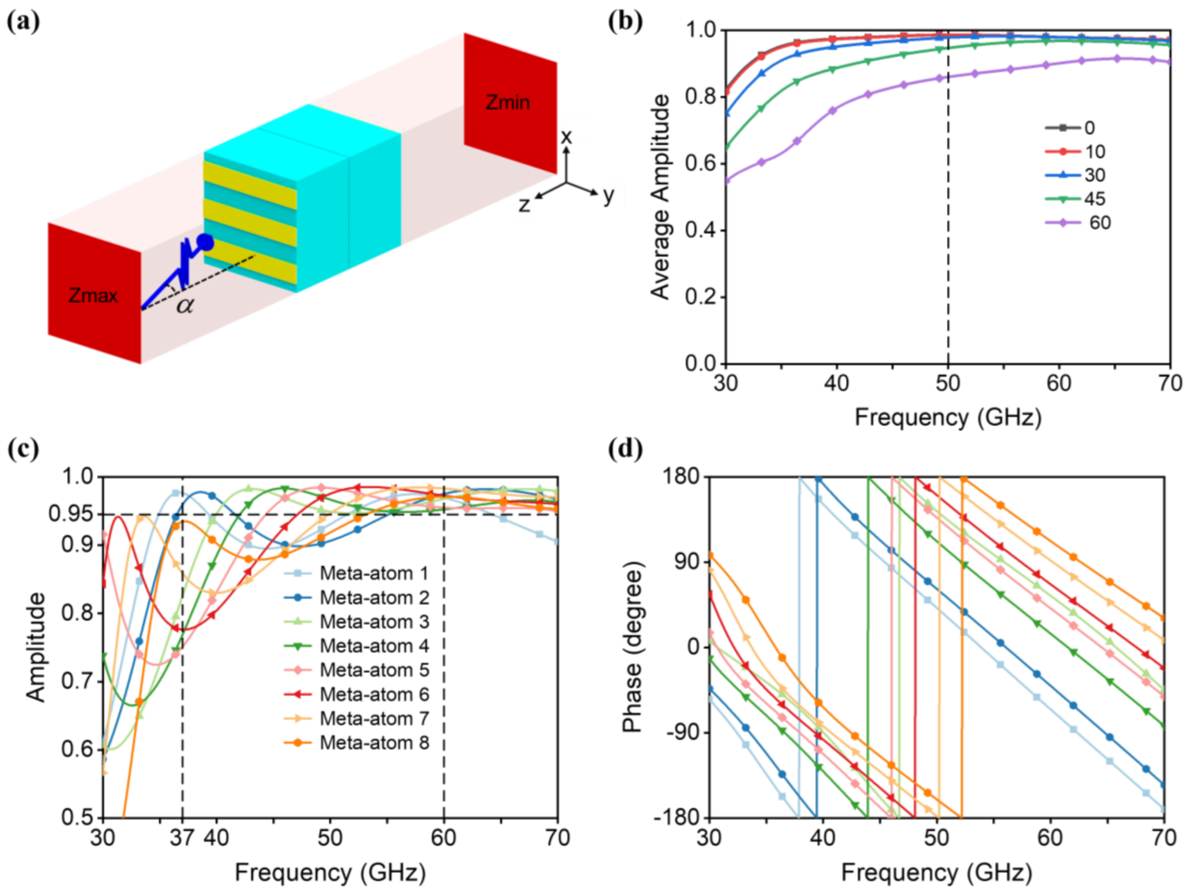

Supplementary Figure S8. Simulation study of the effect of oblique incident angle. (a) Simulation setup. (b) Average transmission amplitude of all the 8 meta-atoms for different incident angles. (c) and (d) are respectively the simulated transmission amplitude and phase shift of the first 8 designed meta-atoms using an incident angle of 45°.

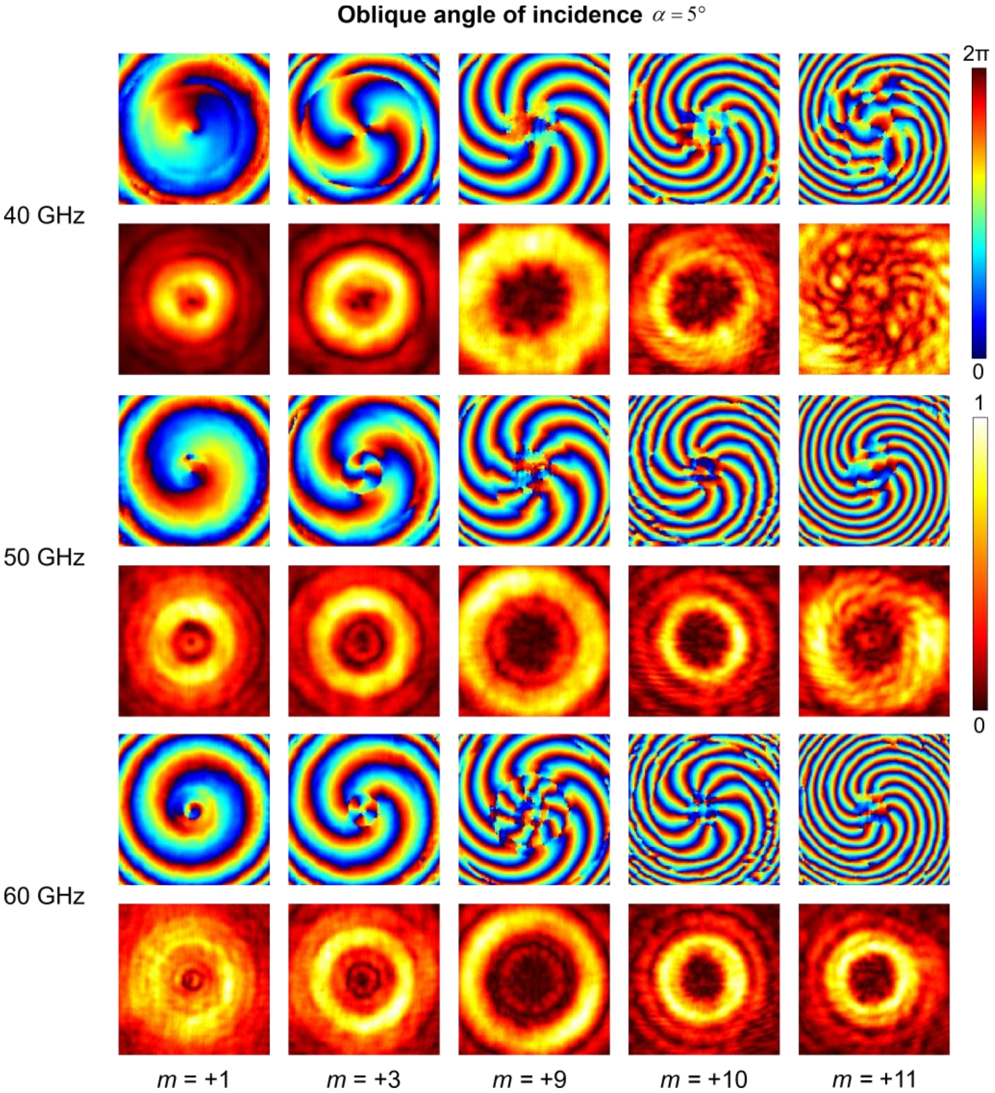

Supplementary Figure S9. Measured phase and amplitude results of 5 example vortex modes using an incident angle of 5°.

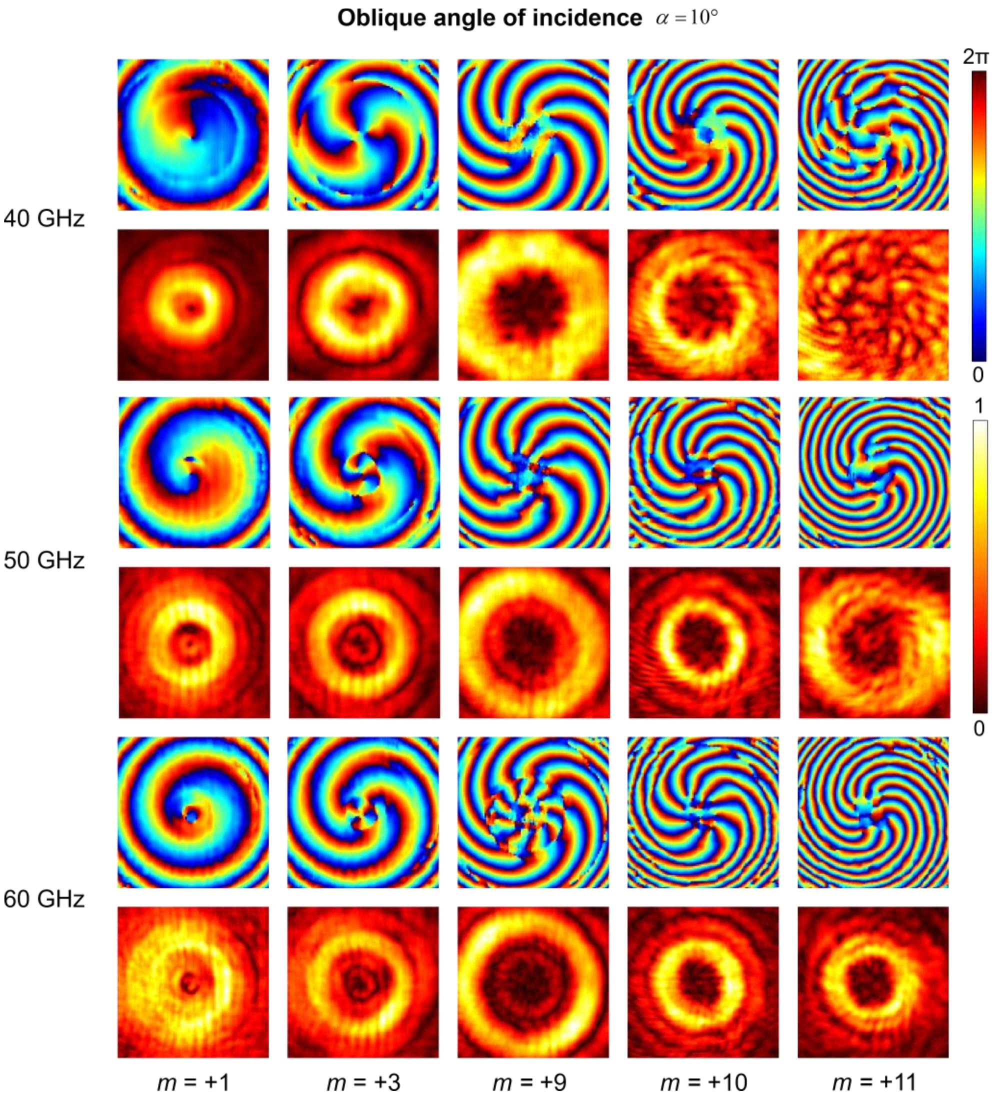

Supplementary Figure S10. Measured phase and amplitude results of 5 example vortex modes using an incident angle of 10°.

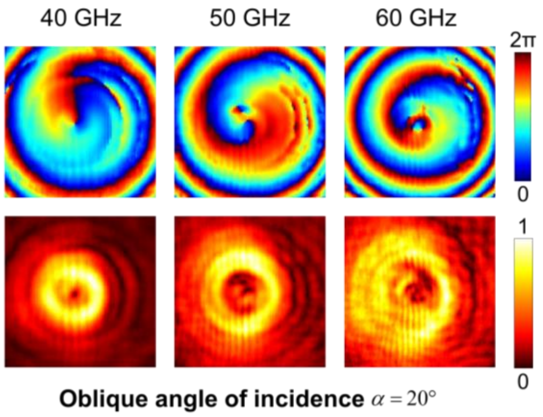

Supplementary Figure S11. Measured phase and amplitude results of vortex mode $m = +1$ using an incident angle of 20°.

Supplementary Note S10. Study of the thickness of substrate layer

The influence of thickness change of the F4B layer is also studied by unit cell simulations. The thickness of the F4B layer is determined according to the Fabry-Perot resonance frequency

$$t = \frac{kc}{8nf}(k = 1, 3, 5, \ldots)$$

where n represents the refractive index of the dielectric layer. Therefore, thickness change of the F4B layer will affect the Fabry-Perot resonant frequency and associated bandwidth. Since the thickness of the applied F4B layer in fabrication generally has an error within ±0.05 mm, we conduct simulations to evaluate the effects of such error. The results given in Figure S12 suggest that good amplitude and phase tuning can still be obtained. But the operating bandwidth tends to shift. To be specific, the operating bandwidth shifts to higher frequencies for a F4B layer while to lower frequencies for a F4B layer, which agrees well with the above equation.

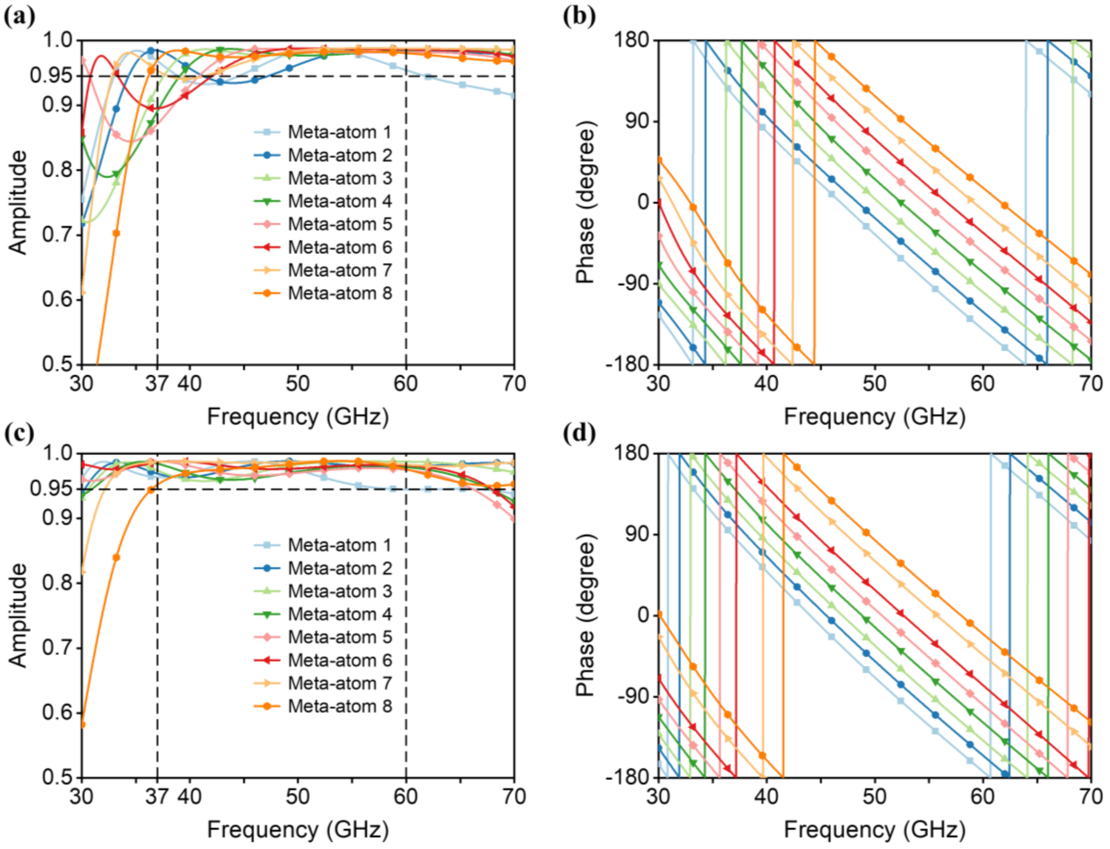

Supplementary Figure S12. Simulation study using different thickness of the F4B substrate layer. (a) and (b) are respectively the simulated transmission amplitude and phase shift of the first 8 designed meta-atoms using thickness $t = 0.45$ mm. (c) and (d) are respectively the simulated transmission amplitude and phase shift of the first 8 designed meta-atoms using thickness $t = 0.55$ mm.
